# Comparing stapler and sutured mesh fixation techniques for laparoscopic TAPP repair: a study on chronic groin pain on 3-year follow-up

**DOI:** 10.1007/s13304-024-01754-1

**Published:** 2024-02-07

**Authors:** Amro Elhadidi, Ahmed Negm, Ashraf Shouma

**Affiliations:** General Surgery Department, Faculty of Medicine, Mansoura University, Mansoura, 35111 Egypt

**Keywords:** Unilateral groin hernia, Stapled mesh fixation, Sutured mesh fixation, Laparoscopic procedure, Postoperative groin pain

## Abstract

Trans-abdominal preperitoneal (TAPP) and totally extraperitoneal (TEP) repairs are the available surgeries for inguinal hernias, with both methods of laparoscopic repairs requiring mesh applications. This study analyzes the correlation between sutured versus stapler mesh fixation in a laparoscopic TAPP for unilateral groin hernia regarding chronic pain during 3-year follow-up. A total of 130 patients with laparoscopic hernia undergoing TAPP repair were randomized into 2 groups based on their fixation technique—one with sutures and the other with stapler. Postoperative complications and chronic groin pain were noted for each technique. Equal number of participants was present in the stapler and suture groups, with the majority having an ASA score of one. The mean age was 42.50 ± 13.86 years, and the body mass index (BMI) was 27.47 ± 5.88. The stapler group presented a shorter mean operative time than the suture group. However, the stapler group had a significantly higher mean VAS score than the suture group. Most participants in the suture group (89.2%) had LOS for 1 day, while a 2-day LOS was significantly higher in the stapler group (12.3%) than in the suture group (9.2%). No patient reported mesh erosion, conversion, recurrence, testicular atrophy, and mesh infection. Early postoperative pain was more in stapler group along with long hospital stay, but both were non-significant. Chronic postoperative pain results and recurrence incidences over 3-year follow-up were also similar. Re-admission rates were minimal, no significant complications occurred.

## Introduction

Approximately 75% of all wall hernias of the abdomen can be attributed to inguinal hernias in both genders, with a 3% lifetime prevalence in women and 27% in males [1]. The two basic laparoscopic procedures for groin hernia repair are the transabdominal preperitoneal (TAPP) and totally extraperitoneal (TEP) methods, both of which require a mesh placement [2]. The mesh is generally composed of polypropylene, an inert, synthetic polymer that does not produce aberrant inflammation. Lightweight and pliable, the mesh is designed to avoid impeding spatial mobility or local structures. Partially dissolvable fibrin adhesives or sutures (or both) can support the mesh, with a stronger seal effectively produced by the glue [3]. In each instance, the repair method is determined by various variables, including the hernial defect’s condition, the surgeon’s choice, and the patient’s characteristics.

Inguinal hernias can be fixed by suturing or applying a prosthetic mesh within an abdominal wall layer to conceal the weakness through open surgeries or laparoscopic methods [4]. Laparoscopic repair has been linked to less postoperative discomfort and a quicker return to regular everyday activities [5]. Even though most laparoscopic inguinal hernia repairs are already done as day operations with excellent results, morbidity in the shape of persistent groin discomfort and hernia recurrence remains a major issue. The total prevalence of moderate to severe postoperative chronic pain is approximately 10% to 12% [6–8], and it can have a major impact on a person’s quality of life. As reported, recurrence following laparoscopic hernia surgery has been found to be 2.5% [9].

Sutures or tacks are commonly used to hold the prosthetic mesh in position during an inguinal hernia surgery. The peritoneum is secured with sutures or tacks in TAPP procedures. These mesh fixing or peritoneal closure methods may add to persistent pain after surgery, owing to nerve irritation or entrapment [10]. Intraoperative pain reduction techniques encompass mesh nonfixation or the application of nonmechanical mesh fixation techniques other than tacking or suturing, which might be less damaging to the local tissue and far less likely to trigger local nerve impingement. Self-fixing meshes and adhesive are examples of non-mechanical techniques. Similarly, suturing the peritoneum may be less painful than using tacks, culminating in less postoperative discomfort [11].

Endoscopic hernia surgery has higher risks than open surgery, with complications including urinary retention, seroma/hematoma, testicular pain/swelling, mesh infection, neuralgia, and recurrence. Seromas are common after laparoscopic surgery, but can heal in 4–6 weeks. To prevent seroma formation in direct hernias, sac dissection was limited and secured it to the pelvic bone. Also, excessive separation of the sac can cause testicular swelling. Strict aseptic measures are necessary to prevent mesh inflammation, and appropriate antibiotics should be administered before surgery [12].

The objective of this study is to investigate the correlation between sutured mesh and stapler fixation in laparoscopic surgery for preperitoneal inguinal hernia treatment, specifically for unilateral groin hernia. The study aims to assess the outcomes in terms of chronic groin pain andpatient discomfort during 3-year follow-up period.

## Method

### Study design

This study compares the use of sutures versus stapler for mesh fixation during TAPP in 130 patients with non-complicated oblique unilateral inguinal hernia. Randomization of patients was performed through a computer-generated schedule, and the results were sealed into envelopes. A nurse not engaged in the study opened the envelopes in the operating room. The study’s key objective was to compare the two techniques’ pain levels on long-term follow-up. The study’s methodology was designed to ensure randomization and minimize bias.

### Location

The study was conducted at Mansoura University hospitals from January 2021 to December 2022.

### Patient recruitment

Patients were recruited consecutively through surgical outpatient clinics. Informed consent for the intervention and study participation was obtained from all patients for participation and intervention in the study.

### Inclusion criteria

Patients above 18 years with non-complicated oblique unilateral inguinal hernia were included in the study. Radiological assessment including abdominal and groin ultrasound to detect the type of hernia and associated abdominal pathology.

### Exclusion criteria

Patients with severe systemic comorbidities including cardiorespiratory and end-stage renal disease, recurrent cases, bilateral cases, and patients with a history of multiple abdominal surgeries were excluded from the study.

### Data collection

Clinical data were collected from medical records, investigations, and operative details. Follow-up was conducted either in the outpatient clinic or through telephone contacts.

### Techniques used

The procedure started by creating carbon dioxide pneumoperitoneum through a small periumbilical incision using closed Veress needle technique. A 10 mm telescope trocar was inserted and the insufflation pressure was kept up to 10–12 mm Hg. Other two 5 mm trocars were placed in bilateral lateral position in the lower abdomen. The procedure start with a thorough abdominal exploration. A lazy S or J transverse peritoneal incision was made 2 cm above from the upper margin of the hernia defect.

A large preperitoneal space was created dissecting both spaces; space of Bogros and space of Retzius till all the medial and lateral inguinal fossae to be dissected including delineation of symphysis pubis, Hesselbach’s triangle making sure not to injury any corona mortis vessels. The hernia sac then dissected from ductus deferens and spermatic vessels. Dissection is the continued till a floppy peritoneal reflection is created and ductus deferens meet the medial umbilical fold. In case of large hernia sac, division was keeping the distal part in place. In case of direct hernia, the transversals fascia is fixed to the pubic bone by sutures or staplers.

**Fig. 1:**
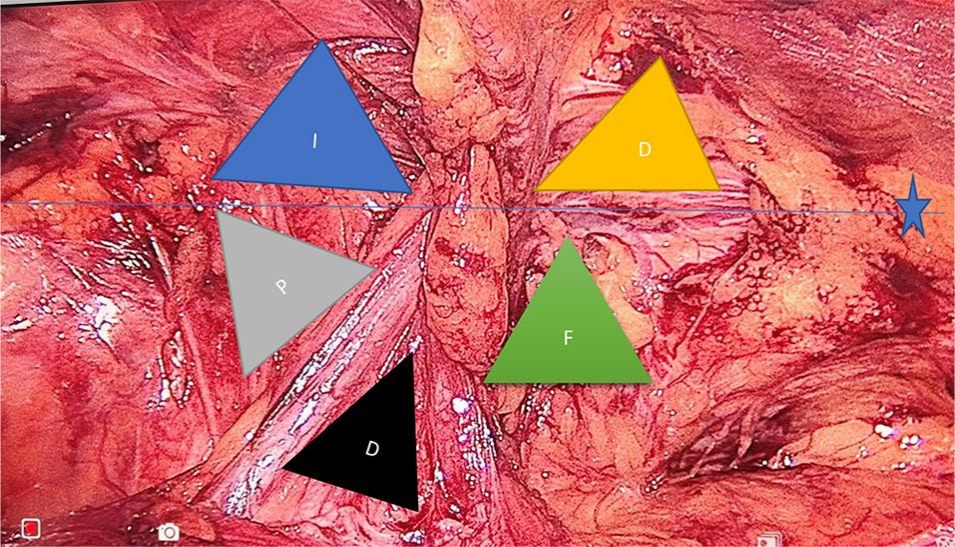
Dissection of five triangles of left hernia (*1* = Indirect, *D* = Direct, *P* = Pain, *D* = Doom, and *F* = Femoral) asterisks denote the inguinal ligament

Once all triangles are dissected (Fig. 1), a large piece of polypropylene mesh (15 × 12 cm in most cases, but the mesh can be tailored to smaller sizes according to patients habitus) was used for TAPP repairs [13]. The polypropylene monofilament mesh was placed all over the hernia orifices. An absorbable tacker was used to fix the mesh around pubic bone, then at maximal lateral position, the maximal three supporting tackers keeping away from inferior epigastric vessels to avoid its injury. The peritoneal flap was closed after decreasing the insufflation pressure to 6 mmHg by 5–6 absorbable tackers. No tackers are allowed in triangles of Doom and Pain. In suturing group, the mesh was fixed by suturing including the area around pubic bone and fixing the mesh superiorly medial and lateral to inferior epigastric vessel with two to three supporting sutures at the lateral fossa by 2-0 prolene sutures. Then the peritoneal edge was closed by either continuous V-lock or vicryl 3-0.

A third-generation cephalosporin was continued in postoperative period. Regarding postoperative inpatient analgesia, non-steroidal anti-inflammatory drug injections were used in the oral analgesia during discharge. Postoperative follow-up was done for all patients at 10 days, one month, 3 months, 6 months, and then yearly during the follow-up periods at outpatient clinic or by telephone contact. All patients are followed up strictly.

The patients are allowed for normal daily activities within the first postoperative weeks and return to work after 2 weeks.

The pain assessment method was based on a pain measurement scale, which included numerical scores from 1 to 10 and a visual analog scale (VAS). The pain levels were categorized as follows: 0 for no pain, 1–3 for mild pain, 4–6 for moderate pain, 7–9 for severe pain, and 10 for the worst imaginable pain.

### Statistical analysis

The recorded data were compiled in a spreadsheet computer program (Microsoft Excel 2010) and exported to the data editor page of IBM SPSS version 22.0 (SPSS Inc., Chicago, Illinois, USA), followed by the application of the chi-square and independent *t* tests. The confidence interval and the *p* value were set at 95% and ≤ 0.05, respectively for all tests.

## Results

The present study included a sum total of 130 patients with laparoscopic hernia repair undergoing TAPP technique. Table 1 presents the study population’s distribution in accordance with independent variables.

**Table 1:** Distribution of study population according to independent variables

Independent variables	Number	Percentage
Group		
Stapler	65	50
Suture	65	50
ASA		
1	100	76.9
2	23	17.7
3	7	7
Total	130	100
Age (Mean ± SD)	42.50 ± 13.86	
BMI (Mean ± SD)	27.47 ± 5.88	

Table 1 shows that there were equal numbers of participants in stapling and suture groups. The majority of them had ASA score of 1. Mean age and BMI were 42.50 ± 13.86 and 27.47 ± 5.88 respectively.

Table 2 shows that compared to the suture group, mean duration of surgery was significantly lesser among stapling group, whereas the mean VAS score of the stapler group was significantly higher compared to the suture group. Only one patient in the stapler group developed port site infection, but the two groups’ difference was non-significant statistically. Also only one patient in stapler group was readmitted during follow-up period for surgical treatment of vaginal hydrocele, but again no statistically significant difference was present in the two groups. Majority of the participants in suture group (89.2%) had LOS for one day while LOS for 2 days was significantly higher among stapling group (12.3%) than in suture group (9.2%). Also, none of them reported mesh Erosion, conversion, recurrence, testicular atrophy and mesh infection.

**Table 2:** Comparative assessment of post-operative outcomes, pain and patient satisfaction among stapler and suture groups

Dependent variables	Stapler group	Suture group	*p* value
Duration of surgery (Mean ± SD)^a^	68.08 ± 12.95	104.52 ± 15.42	0.000*
Follow-up month (Mean ± SD)^a^	32.57 ± 9.19	33.31 ± 8.34	0.632
VAS pain score (Mean ± SD)^a^	3.29 ± 2.28	2.31 ± 1.94	0.009*
Early post-operative outcomes			
Urinary retention^b^ *n* (%)			
No	58 (89.2)	57 (87.7)	0.784
Yes	7 (10.8)	8 (12.3)	
Hematoma^b^ *n* (%)			
No	65 (100)	63 (96.9)	0.154
Yes	0	2 (3.1)	
Seroma^b^ *n* (%)			
No	62 (95.4)	65 (100)	0.080
Yes	3 (4.6)	0	
Nausea and Vomiting^b^ *n* (%)			
No	62 (95.4)	63 (96.9)	0.648
Yes	3 (4.6)	2 (3.1)	
Chest and Pleuritic pain^b^ *n* (%)			
No	62 (95.4)	57 (87.7)	0.245
Yes	3 (4.6)	8 (12.3)	
Ileus^b^ *n* (%)			
No	64 (98.5)	65 (100)	0.315
Yes	1 (1.5)	0	
Port-site infection^b^ *n* (%)			
No	64 (98.5)	54 (83.1)	0.315
Yes	1 (1.5)	0	
Late post-operative outcomes			
Readmission^b^ *n* (%)			
No	65 (98.5)	65 (100)	0.315
Yes	1(1.5)	0	
Pain requiring additional analgesia^b^ *n* (%) in the admission time			
No	58 (89.3)	63 (97)	0.965
Yes	7 (10.7)	2 (3%)	
Length of Hospital Stay (LOS)^b^ *n* (%)			
1 day	56 (86.2)	58 (89.2)	0.049*
2 days	8 (12.3)	6 (9.2)	
3 days	1 (1.5)	1 (1.5)	
Conversion^b^ *n* (%)			
No	65 (100)	65 (100)	–
Yes	0	0	
Mesh erosion^b^ *n* (%)			
No	65 (100)	65 (100)	–
Yes	0	0	
Recurrence^b^ *n* (%)			
No	65 (100)	65 (100)	–
Yes	0	0	
Mesh infection^b^ *n* (%)			
No	65 (100)	65 (100)	–
Yes	0	0	
Chronic pain^b^ *n* (%) (during the follow up peroid)			
No	60 (92.3)	63 (96.9)	0.244
Yes	5 (7.7)	2 (3.1)	
Testicular atrophy^b^ *n* (%)			
No	65 (100)	65 (100)	–
Yes	0	0	
Total	65 (100)	65 (100)	

^a^Independent *t* test

^b^Chi-square test

*Indicates statistically significant difference

## Discussion

Although recurrent and bilateral inguinal hernias have been the main reasons influencing the choice for laparoscopic inguinal hernia repairs, the latter has become a viable choice for inguinal hernia repair. With expanding expertise on laparoscopic methods, it is now effectively applied to repair primary/unilateral inguinal hernias. The laparoscopic method’s potential advantages include faster surgical healing and a lower risk of prolonged groin pain [14].

The worldwide groin hernia management guideline suggests mesh-based repair techniques for individuals with symptomatic inguinal hernias, which can either be laparoscopic inguinal hernia repair (LHR) or open inguinal hernia repair (OHR) [14]. Fewer postoperative pain, paresthesia, chronic groin pain issues, higher patient-reported level of satisfaction, reduced surgical wound problems, shorter hospital accommodation, sooner return to routine actions, and fewer skipped work hours are all major benefits of the former technique over the latter one [15–17].

Based on the set premise, the present study was undertaken to find the correlation between stapler and sutured mesh fixation in a laparoscopic repair of preperitoneal inguinal hernia for unilateral groin hernia regarding chronic groin pain. The chronic groin pain is mostly related to nerve injuries and tissue inflammation. Linderoth et al [18] reported an incidence of chronic groin pain and painful neuralgia can be reached up to 5% of patient undergone laparoscopic repair. This may attributed to mesh fixation technique either absorbable or metal tackers [18]. Some study showed the superiority of non-mesh fixation or mesh fixation by means of histoacryl techniques, but the availability of such products may be insufficient in many institutes adding the higher risk of hernia recurrence [19].

The most common vascular injury during laparoscopic TAPP is inferior epigastric vessel injuries mostly during peritoneal dissection or tacks and sutures applications. Other big named vessels like external iliac vessels almost necessitating conversion with assistance of vascular surgeons. In the present study, only one vascular injury was occurred during dissection of Ritzus space with bleeding from Corona Mortis, which is a variant vascular anastomosis in between the external iliac artery or inferior epigastric artery and obturator artery. The injury was managed urgently with inserting a 10 mm trocar instead of the 5 mm one and gauze compression applied for 5 min then the procedure completed safely [20–22]. In patients with Ehlers–Danlos syndrome, particularly in men, inguinal hernia formation is a common occurrence. Quarto et al. [23] published a study highlighting the successful use of the robotic transabdominal preperitoneal procedure (TAPP) in the repair of inguinal hernias in patients with Ehlers–Danlos syndrome. This study emphasizes the feasibility of robotic technology, emphasizing its benefits, such as shorter recovery times and improved cosmetic outcomes, while also adding valuable insights to the broader context of inguinal hernia repair [23]. There were no Ehlers–Danlos syndrome patients reported in our study, and robotic surgery was not a routine practice at our institution. However, given the promising results obtained when using robotic transabdominal preperitoneal procedure (TAPP) for inguinal hernia repair in such patients, future research should focus on comparative studies between robotic TAPP and laparoscopic TAPP on larger patient cohorts to better understand the potential benefits and limitations of these surgical approaches.

Every surgical subject is interested in preventing wound infections upon surgical interventions in order to enhance patient care quality. Surgical-site infections are among the most prevalent nosocomial illnesses, and they frequently result in longer hospital stays and higher expenses [24, 25]. Only one patient in the stapler group became infected at the port site, but the two groups’ difference was statistically insignificant. Additionally, only one patient in the stapling group was readmitted for surgical treatment of a vaginal hydrocele throughout the study period although no statistically significant difference was present between the two groups. In a trial of patients with inguinal hernia, five patients acquired vaginal hydrocele, which was discovered at the three-month follow-up appointment and proceeded to increased size and pain over time, necessitating surgery [26].

Despite being a relatively safe and effective procedure, hernia repair surgery possesses several potential issues and complications that may arise, including recurrence and early postoperative pain. Recurrence of a hernia is one of the most significant issues after surgery. The risk of recurrence depends on several factors, including type and size of the hernia, the surgical technique used, and the patient’s overall health [27]. Recurrence rates vary widely depending on the type of hernia and surgical technique. Mathhews et al. discussed the factors associated with hernia recurrence after laparoscopic and open repair surgeries. The study found that previously repaired hernia was a predictor of recurrence in the open group, but not in the laparoscopic group. Obesity was found to be protective against hernia formation, but a BMI of less than 25 was a risk factor for recurrence in the laparoscopic group. Other factors found to be associated with recurrence include preoperative activity level, influence of a caregiver, ASA class, and recent hernia enlargement. Type of hernia and surgeon experience were also identified as risk factors for recurrence. Mesh-based techniques are found to have a lower recurrence rate compared to tissue repairs [28]. When it comes to chronic pain outcomes, there is no conclusive evidence that mesh-based techniques cause more chronic pain than tissue-based repairs. Laparo-endoscopic repair is found to have advantages in terms of early postoperative pain, analgesic use, and speed of recovery compared to open mesh repair [28].

Laparoendoscopic techniques have advantages over open mesh-based repair in terms of early postoperative pain, analgesic use, and speed of recovery. The HerniaSurge Analysis has reported that chronic pain occurred significantly less often after laparo-endoscopic repair than after Lichtenstein operation [14]. This difference in pain and early recovery led to the recommendation of laparo-endoscopic repair as the preferred operation for primary unilateral inguinal hernia. Robotic surgery has become a viable option for minimally invasive hernia repair and offers advantages, such as reduced postoperative pain associated with mesh fixation and improved ergonomics for surgeons [29]. Additionally, robotic surgery is proving to be valuable in managing complex abdominal wall hernias by providing better visualization and allowing precise wristed movements during procedures. Moreover, the learning curve for robotic hernia repair appears to be less steep compared to laparoscopic techniques, leading to an increase in the use of minimally invasive approaches for hernia surgeries [30]. Anoldo et al. studies 15 patients with simultaneous primary inguinal and umbilical hernias who underwent robotic repair with the da Vinci Xi platform using the dual docking technique. The study found no intraoperative complications and no major complications or recurrences during the follow-up period. These findings imply that the dual docking technique is feasible, reproducible, and results in optimal postoperative outcomes, such as early mobilization and a short length of stay. The research will help surgeons who perform multiquadrant robotic surgery to treat abdominal hernias [31].

It is believed that stapler fixation of mesh during laparoscopic inguinal surgery is essential for preventing recurrence. Mesh fixation, on the other hand, may raise surgery complications and discomfort. The majority of suture group participants (89.2%) had LOS for one day; however, LOS for two days was considerably higher in the stapler group (12.3%) than in the suture group (9.2%). They also did not report mesh erosion, conversion, recurrence, testicular atrophy, or mesh infection. Six studies enrolling a total of 932 patients were included: the mesh was fixed in 463 individuals and not fixed in 469 patients. There was no change in the frequency of recurrence, associated complications or postoperative pain level between the studied groups [32].

The current study demonstrated that there was no significant difference in post-operative chronic groin pain between the sutured and stapler mesh fixation groups. The report was concordant with the ones which indicated that there was no substantial variation in postoperative pain between stapler and sutured mesh fixation for laparoscopic TAPP Repair.

## Conclusion

Chronic post-operative pain results and recurrence incidence were similar in both groups and to those described in the publications. The study demonstrated the fact that the stapler group has more early postoperative pain, but the results were comparable in both groups, and also the same for hospital stay. The re-admission rate was minimal, and no significant complications occurred. Subjective patient evaluations of postoperative pain may be expressed to furnish surgeons with detailed details which can be utilized to identify the optimal type of technique to employ and to more accurately forecast the patient’s postoperative trajectory.

## Data Availability

The availability of data and materials: The datasets used and/or analyzed in this study are available from the author upon reasonable request.
